# Loss of Vascular Endothelial Growth Factor A (VEGFA) Isoforms in Granulosa Cells Using *pDmrt-1-Cre* or *Amhr2-Cre* Reduces Fertility by Arresting Follicular Development and by Reducing Litter Size in Female Mice

**DOI:** 10.1371/journal.pone.0116332

**Published:** 2015-02-06

**Authors:** Kevin M. Sargent, Ningxia Lu, Debra T. Clopton, William E. Pohlmeier, Vanessa M. Brauer, Napoleone Ferrara, David W. Silversides, Andrea S. Cupp

**Affiliations:** 1 Department of Animal Science, University of Nebraska-Lincoln, Lincoln, Nebraska, United States of America; 2 University of California San Diego School of Medicine, San Diego, CA, United States of America; 3 Department of Veterinary Biomedicine, Faculty of Veterinary Medicine, University of Montreal St-Hyacinthe, Québec, Canada; Qingdao Agricultural University, CHINA

## Abstract

Because VEGFA has been implicated in follicle development, the objective of this study was to determine the effects of granulosa- and germ cell-specific VEGFA loss on ovarian morphogenesis, function, and female fertility. *pDmrt1-Cre* mice were mated to floxed VEGFA mice to develop granulosa-/germ cell-specific knockouts (*pDmrt1-Cre;Vegfa* ^-/-^). The time from mating to first parturition was increased when *pDmrt1-Cre;Vegfa* ^-/-^ females were mated to control males (*P* = 0.0008) and tended to be longer for heterozygous females (*P* < 0.07). Litter size was reduced for *pDmrt1-Cre;Vegfa* ^-/-^ females (*P* < 0.007). The time between the first and second parturitions was also increased for heterozygous females (*P* < 0.04) and tended to be increased for *pDmrt1-Cre;Vegfa* ^-/-^ females (*P* < 0.07). *pDmrt1-Cre;Vegfa* ^-/-^ females had smaller ovaries (*P* < 0.04), reduced plasma estradiol (*P* < 0.007), fewer developing follicles (*P* < 0.008) and tended to have fewer corpora lutea (*P* < 0.08). Expression of *Igf1r* was reduced (*P* < 0.05); expression of *Foxo3a* tended to be increased (*P* < 0.06); and both *Fshr* (*P* < 0.1) and *Sirt6* tended to be reduced (*P* < 0.06) in *pDmrt1-Cre;Vegfa* ^-/-^ ovaries. To compare VEGFA knockouts, we generated *Amhr2-Cre;Vegfa* ^-/-^ mice that required more time from mating to first parturition (*P* < 0.003) with variable ovarian size. Both lines had more apoptotic granulosa cells, and vascular staining did not appear different. Taken together these data indicate that the loss of all VEGFA isoforms in granulosa/germ cells (proangiogenic and antiangiogenic) causes subfertility by arresting follicular development, resulting in reduced ovulation rate and fewer pups per litter.

## Introduction

Vascular endothelial growth factor A (VEGFA) is one of the key factors regulating angiogenesis in the ovary [1]. VEGFA stimulates neovascularization, vascular permeability, acts as a survival factor and can also stimulate proliferation of vascular and nonvascular cells [2–4]. An *in situ* study showed the *in vivo* expression of VEGFA in different cell types including theca, cumulus, granulosa and luteal cells in the mouse ovary [5]. Our laboratory has demonstrated that VEGFA and its receptors are localized to pre-granulosa and granulosa cells of all follicle stages and to theca cells and oocytes of advanced-stage follicles in the rat [6]. The *Vegfa* gene can be alternatively spliced to produce angiogenic or antiangiogenic isoforms which have divergent functions from one another: angiogenic isoforms promote vascular development while antiangiogenic isoforms inhibit vascular development [6,7]. To initiate biological effects, VEGFA binds two related tyrosine kinase receptors: FMS-like tyrosine kinase 1 (FLT1, also known as VEGFR1) and kinase insert domain receptor (KDR, also known as VEGFR2 and FLK1). The primary receptor is KDR [8]. KDR is the major mediator of the mitogenic, angiogenic and permeability enhancing effects of VEGFA. KDR is also involved in mediating endothelial cell proliferation, survival and vascular permeability, whereas FLT1 may be inhibitory by sequestering VEGFA, and preventing its interaction with KDR [9]. Roberts *et al*. reported ovarian administration of antibodies to KDR resulted in significant depletion of primordial follicle numbers, whereas anti-FLT1 antibodies did not [10]. Thus, it appears that KDR is critical for follicle progression.

Independent of its angiogenic effects, VEGFA may also be important in the regulation of follicle growth with direct effects on granulosa cells. While rat ovaries treated with VEGFA receptor-tyrosine kinase inhibitor had vascular development reduced by 94%, there were more primordial follicles and fewer early primary, transitional, and secondary follicles compared to controls suggesting a block in follicle progression [6]. This study demonstrated a novel role for VEGFA in the recruitment of primordial follicles into the growing follicle pool, as well as being a potential survival factor for primary and later-stage follicles through vascular-dependent and vascular-independent mechanisms [6]. Further studies from our laboratory demonstrated that treatment with proangiogenic isoform VEGFA_164 or neutralization of antiangiogenic VEGFA isoforms enhanced vascular and follicular development in perinatal rat ovaries [7]. These results suggest that VEGFA proangiogenic isoforms stimulate follicle progression and antiangiogenic isoforms inhibit or arrest follicle development [7]. Furthermore, our laboratory has demonstrated that inhibition of antiangiogenic isoforms stimulates progression of follicles to later stages of development providing further support that their normal function is to inhibit folliculogenesis. Antiangiogenic isoforms were localized to granulosa and theca cells of all stages of follicles in rats [7]. KDR-LacZ-positive staining in the oocytes of secondary follicles in mice further suggests a role of VEGFA directly on oocyte development [11].

Thus, in the current study, we hypothesized that production of VEGFA isoforms (both pro and antiangiogenic) by granulosa cells is critical for normal ovarian morphogenesis and function. Therefore, the objective of the present study was to determine the *in vivo* effects of granulosa (*pDmrt1* and *Amhr2*) and potentially germ cell- (*pDmrt1*) specific VEGFA loss on ovarian function and morphogenesis through conditional knockout mice in which *Vegfa* isoforms are eliminated via either the *pDmrt1* or *Amhr2* promoter.

## Materials and Methods

### Animals

Granulosa and germ cell VEGFA knockout (KO) mice were generated by mating a *Vegfa*-floxed mouse line [12] with porcine doublesex and mab-3 related transcription factor 1 (DMRT1) promoter (*pDMRT1*)-Cre mice [13]. Homozygous *Vegfa*-floxed mice were mated to mice whose genomic DNA genotyped positively for the *pDmrt-1-Cre* allele. Mating these parental lines resulted in F1 progeny, and heterozygous mice from the F1 generation were then mated back to the homozygous *Vegfa* floxed founders to generate the F2 population. Control and homozygous knockout female mice were generated from the F2 generation. We used Cre-negative *Vegfa^-/-^* or *Vegfa* ^*+/-*^ mice as controls and Cre-positive *Vegfa^-/-^* mice as knockouts (KOs) (*pDmrt1-Cre;Vegfa^-/-^*) or Cre-positive *Vegfa* ^*+/-*^ as heterozygotes (Het). *Dmrt1* is expressed in the indifferent mouse gonad at E10.5 in precursor cells that differentiate into Sertoli and granulosa cells [14]. By E13.5, expression is no longer detectable in granulosa cells but is still present within oocytes until E15.5 [14,15]. The *pDmrt1* gene is expressed similarly to mouse *Dmrt1*, as detected by reverse transcriptase (RT)-PCR in developing ovaries, making it a useful means to eliminate *Vegfa* in granulosa and germ cells [13]. In our *pDmrt1-Cre;Vegfa^-/-^* mouse, Cre recombinase activity is driven by the *pDmrt1* promoter which excises exon 3 of the *Vegfa* gene. Exon 3 is present in both proangiogenic and antiangiogenic isoforms; thus, excision results in a reduction in all *Vegfa* isoforms where *pDmrt1* is expressed (S1 Fig.). Ovaries plus oviducts, uteri, kidneys, and adrenals were collected from 8-month-old female mice and weighed in addition to measuring total body weight (n = 11 controls, n = 10 KO). Females were collected in estrus.

Similarly, we generated a transgenic mouse line by crossing the same floxed *Vegfa* mice with mice expressing *Amhr2-Cre*. Expression of the *Amhr2-Cre* transgene was detected as early as 12.5 dpc and continued through adulthood in pre-granulosa cells, granulosa cells and the female reproductive tract [16]. These mice were harvested at approximately 3-months-of-age, earlier than collection of the *Vegfa* x *pDmrt1-Cre* animals (8 months). Reproductive potential of the 8-month-old control animals was not impaired. The mice were harvested at different ages in an attempt to compare reproductive phenotypes resultant from similar cell-specific elimination of VEGFA both early and late in reproductive life.

All animal protocols were approved by the University of Nebraska Institutional Animal Care and Use Committee (IACUC) in accordance with the National Institutes of Health Guide for the Care and Use of Laboratory Animals. All animal models were procured from collaborating investigators with verbal or written MTA agreements. Thus, the corresponding author does not have rights to freely distribute these mouse lines.

### Genotyping

Genomic DNA was extracted from tail samples according to previously reported methods [17]. RNA was extracted from whole ovaries and reverse transcribed to cDNA to be tested for *Cre* expression at the level of the gonad. Animals were removed from analysis if results differed from genomic DNA genotyping. Primer sequences are supplied in Table 1.

**Table 1:** Sequences for primers used to conduct conventional and quantitative PCR.

PCR	Gene	Sequence	Accession Number
Conventional	*pDmrt1*(forward)	5’-AGCAGAGGCTTCCTTCGACTT-3’	AF216651
	*Cre* (reverse)	5’-AGTGAACGAACCTGGTCGAAA-3’	
	muVEGF419.F	5’-TCCGTACGACGCATTTCTAG-3’	
	muVEGF567.R	5’-CCTGGCCCTCAAGTACACCTT-3’	
qPCR	*Foxo3a*	5’-GCAAAGCAGACCCTCAAACTG-3’	NM_019740
		5’-TGAGAGCAGATTTGGCAAAGG-3’	
	*Amh*	5’-TCCTACATCTGGCTGAAGTGATATG-3’	NM_007445
		5’-CAGGTGGAGGCTCTTGGAACT-3’	
	*Gdf9*	5’-GCCGGGCAAGTACAGCC-3’	NM_008110
		5’-TTTGTAAGCGATGGAGCCG-3’	
	*Igf1r*	5’-CAGATGGCTGGAGAGATTGCA-3’	NM_010513.2
		5’-GTGGACGAACTTGTTGGCATT-3’	
	*Fshr*	5’-TCCGCAGGGACTTCTTCGT	NM_013523.3
		5’-AATCTGGGCTTGCACCTCAT	
	*Bmp2*	5’-TGTCCCCAGTGACGAGTTTCT-3’	NM_007553.3
		5’-CCTGTATCTGTTCCCGGAAGAT-3’	
	*Sirt1*	5’-AAACCTCCACGCCCACAA-3’	NM_019812.2
		5’-GAAGAGGTGTTGGTGGCAACTC-3’	
	*Sirt6*	5’-AGCCCCCCGTGCATCT-3’	NM_181586.3
		5’-TGTTGGGCTTGGACTTATACGA-3’	

### Fertility Trials

Cyclic female mice were paired with control males when they were approximately 2–4 months-of-age and remained together until they were six to eight months-of-age for both *pDmrt1-Cre;Vegfa^-/-^* and *Amhr2-Cre;Vegfa^-/-^* *mice*. Control, Het and KO females were all paired with control males of proven fertility. The time from when males and females were placed together in cages to the time of parturition was measured in days. We also determined the number of pups per litter. Het females were removed from the *Vegfa* x *Amhr2-Cre* analysis due to ectopic *Cre* expression.

### Fixation, Embedding, Staining, and Immunohistochemistry (IHC)

Ovaries were fixed at room temperature in Bouin’s solution, embedded in paraffin and sectioned at 5 μm according to standard procedures [6]. The rabbit polyclonal IgG VEGFA primary antibody was raised against a peptide corresponding to amino acids 1–140 of human VEGFA (catalog number: sc-507, Santa Cruz Biotechnology, Santa Cruz, CA). As a pan-antibody, it targeted both proangiogenic and antiangiogenic isoforms to confirm presence of VEGFA isoforms in *KO* and control mouse ovaries. The antibody was diluted 1:100 in 10% normal goat serum (NGS). As a negative control, serial sections were processed without primary antibody. Biotinylated goat anti-rabbit secondary antibodies were diluted 1:300 in 10% NGS and were used with each primary antibody in this study (catalog number: BA-1000, Vector Laboratories, Burlingame, CA). The secondary antibody was detected using aminoethyl carbazole (AEC) chromagen substrate solution (Invitrogen, Carlsbad, CA). Similar immunohistochemistry protocols were used for additional primary antibodies. The cytochrome P450, family 11, subfamily a, polypeptide 1 (CYP11A1) primary antibody (catalog number: ab78416; Abcam, Cambridge, Mass., USA) was diluted 1:200 in 10% normal goat serum in PBS) and was used to quantify the number of corpora lutea present.

### Counts of Follicles and Corpora Lutea

In order to determine the number of follicles per ovary, three images per ovary were counted by two technicians whose counts were averaged, and means were compared between *Vegfa* x *pDmrt1-Cre* control (n = 8) and KO (n = 9) ovaries as well as for *Vegfa* x *Amhr2-Cre* control (n = 4) and KO (n = 5) ovaries. One image was taken of the same ovary sectioned at representative depths- the most proximal and distal sections as well as the centermost section. Follicles were staged according to previously reported methods [6,7]. Stage 0 or primordial follicles consisted of an oocyte surrounded by a single layer of squamous granulosa cells. Stages 1–5 were grouped together as developing follicles. Stage 1 follicles were characterized by an oocyte surrounded by a single layer of a mixture of squamous and cuboidal granulosa cells. To be classified as Stage 2, all granulosa cells were cuboidal. Two or more layers of squamous granulosa cells had to present to be considered a Stage 3 follicle. Stage 4 follicles had a visible theca cell layer as well as an antrum. Stage 5 follicles had a large antrum and appeared to be ready to ovulate. Corpora lutea (CL) were evidenced by positive staining for the steroidogenic enzyme CYP11A1. The number of CL on one ovary per animal was counted and compared between *Vegfa* x *pDmrt1-Cre* control (n = 8) and KO ovaries (n = 9). Follicles were counted at 200x magnification.

### Quantitative RT- PCR (qPCR)

One ovary was collected from each animal for RNA extraction, reverse transcription to cDNA and quantitative Real-Time PCR (qPCR) analysis according to previous protocols [6,17]. Primers and probes were designed with Primer Express 3.0 software (Applied Biosystems) and synthesized at Integrated DNA Technologies, Inc. (Coralville, IA). Amplification of genes that required SYBR Green to measure quantification was also plotted on a dissociation curve to ensure primer dimerization did not occur. We designed probes for two genes: BCL2-associated X protein (*Bax*) and B cell leukemia/lymphoma 2 (*Bcl2*). Primer and probe sequences for each gene are found in Table 1. Primers were designed for *Foxo3a, Igf1r, Fshr, Bmp2, Sirt1, Sirt6, Amh*, and *Gdf9*. Messenger RNA abundance was normalized to expression of the housekeeping gene, glyceraldehyde-3-phosphate dehydrogenase (*Gapdh*) and represented as a fold change relative to the mean control value (set at a value of 1). The expression of *Gapdh* was determined using a VIC probe and primer kit (Taqman Rodent GapDH Control Reagents; Applied Biosystems; Catalog #4308313). Values were expressed as means±SEM.

### Hormone Assays

Trunk blood was collected at the time of euthanasia and prepared for ELISA according to routine methods [17]. Plasma estradiol concentrations were determined by ELISA kit (Catalog #1920, Alpha Diagnostics International Inc., San Antonio, TX). Samples remained undiluted and were run according to the manufacture’s direction. All unknown values were plotted against a standard curve. Assay sensitivity was 10 pg/ml. The progesterone concentrations were determined by ELISA kit (Catalog #1860, Alpha Diagnostics International Inc.). Assay sensitivity was 0.2 ng/ml. Testosterone concentrations were determined by a testosterone ELISA kit (catalog number: 1880; Alpha Diagnostics International Inc., San Antonio, TX) [18]. Assay sensitivity was 0.125 ng/ml.

### Immunofluorescence

Ovaries were processed for immunofluorescence as described for IHC and according to previous methods in our laboratory [17] with a rabbit polyclonal primary antibody to cleaved Caspase 3 (CASP3; Asp 175; catalog number: #9661; Cell Signaling, Danvers, MA). An anti-rabbit FITC secondary antibody (Alexa Fluor 488; catalog number: #4412S; Cell Signaling) was added to bind the cleaved CASP3 primary antibody, and it was diluted 1:1000 in 3% bovine serum albumin (BSA) in PBS-Tween. A goat antibody to VE-CADHERIN (catalog number: #sc-6458; Santa Cruz Biotechnology, Inc.) was also used to visualize vasculature. To detect expression of VE-cadherin, we used an anti-goat Texas Red secondary antibody (Alexa Fluor 594; catalog number: #A-21223; Invitrogen/Life Technologies, Grand Island, NY). We also stained ovaries from *Vegfa* x *Amhr2-Cre* control and KO (*Amhr2-Cre;Vegfa^-/-^*) females to increase the number of representative images since they have a similar mechanism of eliminating *VEGFA*. Two images were taken at 200x of both control (n = 3) and KO (n = 4) ovaries. Both primary antibodies were diluted 1:50 in 3% BSA (catalog number: #A3311; Sigma-Aldrich Corp., St. Louis, MO) in PBST, and both secondary antibodies were diluted 1:1000 in 3% BSA.

### Statistical Analysis

Fertility data were analyzed by one-way ANOVA followed by t-test using SAS software (SAS Institute, Cary, NC). All other data were analyzed using the Dunnett’s procedure in JMP (SAS Institute). Differences in data were considered statistically significant when *P* < 0.05. Data were considered tending to be different if 0.05 > *P* < 0.1. All treatments were compared relative to controls. Gene expression data were tested for normality, and data that were not normally distributed were square rooted using SAS. Means reflect the original data, but *P*-values are representative of the Dunnett’s test following transformation.

## Results

### *pDmrt1-Cre;Vegfa^-/-^* mice were subfertile and had reduced ovarian weight

**Fig 1:**
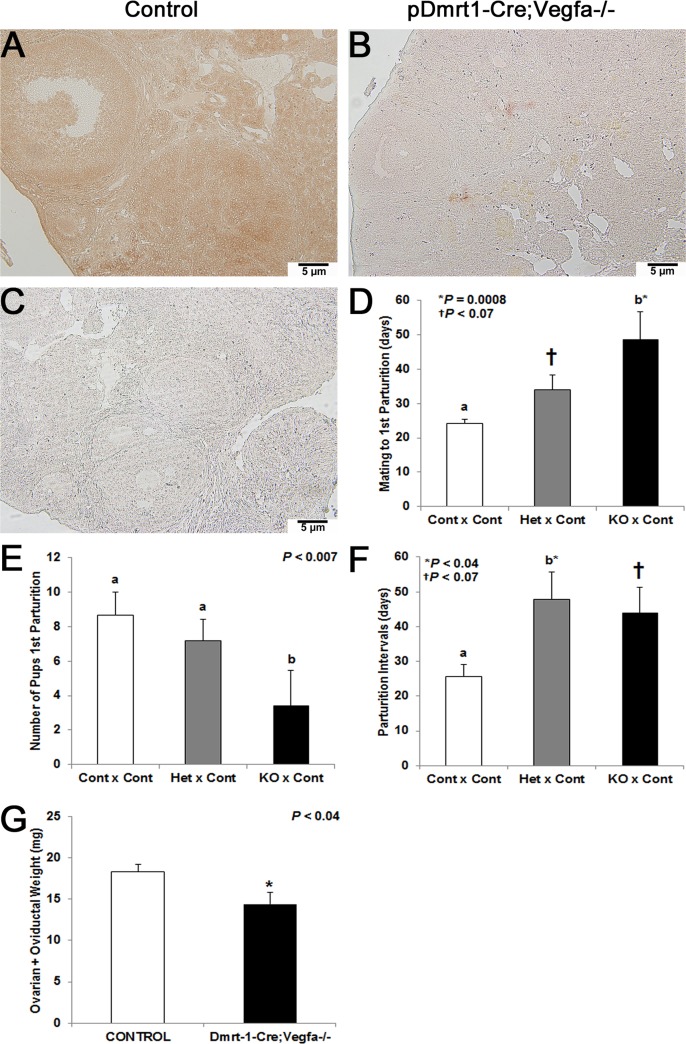
Immunohistochemistry with panVEGFA antibody to confirm VEGFA knockdown via *pDmrt1-Cre* and female fertility: 200x magnification in a *pDmrt1-Cre;Vegfa^-/-^* ovary (B) and a control ovary (A). Negative control ovaries were processed without primary antibody (C). Females are named first in each fertility trial pairing (Control, Het, KO) for *Vegfa* x *pDmrt1-Cre* and were broken into mating to first parturition (D), the number of pups born at the first parturition (E) and the number of days from the first parturition to the second (F). For the Control x Control, Het x Control, and KO x Control pairs in both (D) and (E), n = 12, 10, and 5, respectively. For the Control x Control, Het x Control, and KO x Control pairs in (F), n = 4, 4, and 5, respectively. Ovarian plus oviductal weight (g) is presented in KO vs control females (G), and n = 10 and 11, respectively. Results represented by either different letters or a * were significant when *P* < 0.05, and a † denoted data that tended to be significant (0.05 < *P* < 0.1).

Immunohistochemistry with a pan-VEGFA antibody was performed to confirm the deletion of both pro- and antiangiogenic isoforms of VEGFA in KO ovaries at 8 months-of-age. The intensity of VEGFA-positive staining was dramatically reduced in ovaries taken from *pDmrt1-Cre;Vegfa^-/-^* females (Fig. 1B) compared to controls (Fig. 1A). Sections processed without primary antibody did not stain and served as negative controls (Fig. 1C).

Mice were placed in mating pairs as follows: 1) control females with control males (Contl x Contl, n = 12), 2) heterozygous females with control males (Het x Cont, n = 10), and 3) KO females with control males (KO x Contl, n = 5). Het x Cont mating pairs from *Vegfa* x *pDmrt1-Cre* mice tended to take 10 days longer to get pregnant during the first mating to parturition interval (*P* < 0.07), and KO females took significantly longer to get pregnant when mated to control males (*P* = 0.0008) compared to Cont x Cont pairs (Fig. 1D). While there were no differences in the number of pups born in the first litter for Het x Control (*P* = 0.32), there were fewer pups born to KO x Cont mating pairs (*P* < 0.007) compared to Cont x Cont matings (Fig. 1E). Finally as seen in Fig. 1F, the number of days between the first and second parturitions for Het x Control pairs was increased (*P* < 0.04) and tended to be increased for KO x Cont mating pairs (*P* < 0.07) compared to the length for Cont x Cont mating pairs. Females were collected at the end of the fertility trial, and the combined weight of ovaries and oviducts from *pDmrt1-Cre;Vegfa^-/-^* females was significantly decreased compared to control females (*P* < 0.04, Fig. 1G). Control mice at the end of the fertility trial were still reproductively viable while the KO mice were less so, as indicated by the preceding data.

### Plasma estradiol concentrations were reduced in *pDmrt1-Cre;Vegfa^-/-^* *mice*

**Fig 2:**
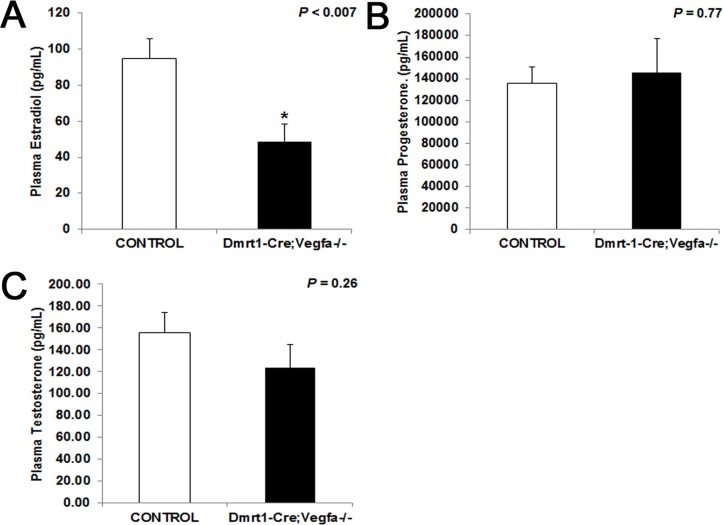
Plasma hormone concentrations in *Vegfa* x *pDmrt1-Cre* females. Concentrations of plasma E_2_ (A), progesterone (B) and testosterone (C) in KO (n = 7–10) compared to controls (n = 9–11). Results were significant when *P* < 0.05 and were represented with a *.

Plasma 17β-estradiol in blood collected at the end of the fertility trial was reduced by almost half in *pDmrt1-Cre;Vegfa^-/-^* females (*P* < 0.007) compared to control females (Fig. 2A). There was no significant difference in plasma progesterone concentrations between the *pDmrt1-Cre;Vegfa^-/-^* (*P* = 0.77) and control females (Fig. 2B). There was also no difference in plasma testosterone concentrations in *pDmrt1-Cre;Vegfa^-/-^* females (*P* = 0.26) compared to control females (Fig. 2C).

### There were fewer developing follicles and a tendency for a reduction in CL number in *pDmrt1-Cre;Vegfa^-/-^* *ovaries*

**Fig 3:**
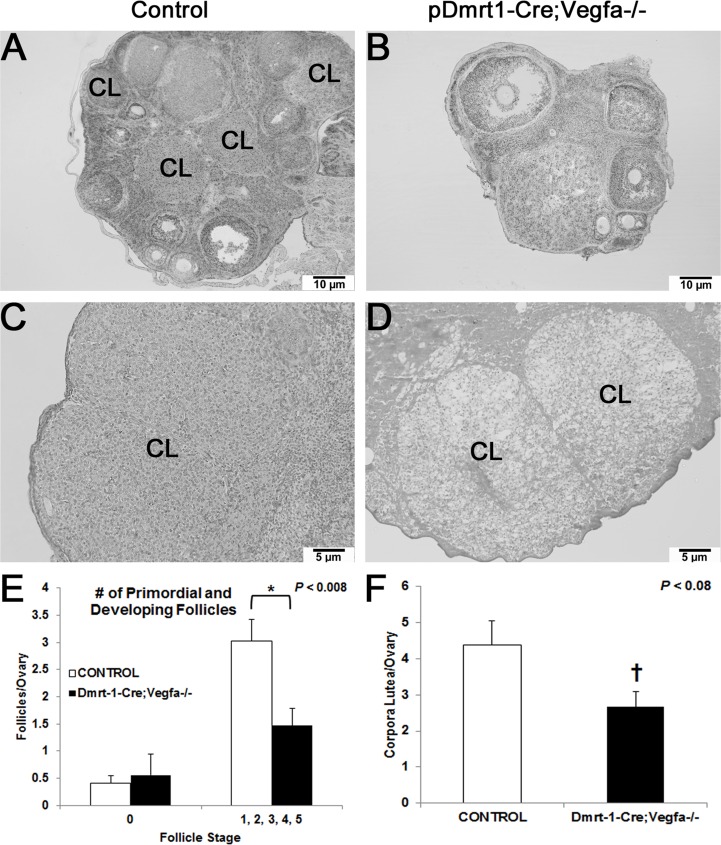
*pDmrt1-Cre;Vegfa^-/-^* and control ovary histology. Hematoxylin-eosin staining of ovarian cross-sections of 200x control (A), 200x control (C), 200x KO (B), and 200x KO (D). The 200x images depict varied CL morphology between a control and KO. Some of the ovarian structures are labeled: corpus luteum (CL) and the stage of follicle (1–4). The number of primordial follicles (stage 0) and developing follicles (stages 1–5) were counted and compared between controls (n = 8) and KO (n = 9) (E). The number of CL per ovarian section was counted based on positive staining for CYP11A1 and compared between controls (n = 8) and KO (n = 6) (F). Results were significant when *P* < 0.05 and were represented with a *, and a † denoted data that tended to be significant (0.05 < *P* < 0.1).

While the number of primordial follicles (Stage 0) was not different per ovary between *pDmrt1-Cre;Vegfa^-/-^* and control females, *pDmrt1-Cre;Vegfa^-/-^* females had half as many developing follicles (Stages 1–5) per ovary (*P* < 0.008) as control females (Fig. 3E). Fewer later stage follicles can be seen on the accompanying 100x images of a *pDmrt1-Cre;Vegfa^-/-^* ovary (Fig. 3B) compared to a control ovary (Fig. 3A), and the *pDmrt1-Cre;Vegfa^-/-^* ovaries appear smaller than controls. In addition, the number of CL per ovary on *pDmrt1-Cre;Vegfa^-/-^* ovaries (*P* < 0.08) tended to be reduced compared to control mice (Fig. 3F). At 200x, the morphology of two CL in a representative KO ovary appeared to have fewer and less densely compacted luteal cells compared to a control (Fig. 3C, D).

### *pDmrt1-Cre;Vegfa^-/-^* female ovaries had reduced *Igf1r, Fshr, Sirt6* and*Foxo3a* mRNA at 8 months of age

**Fig 4:**
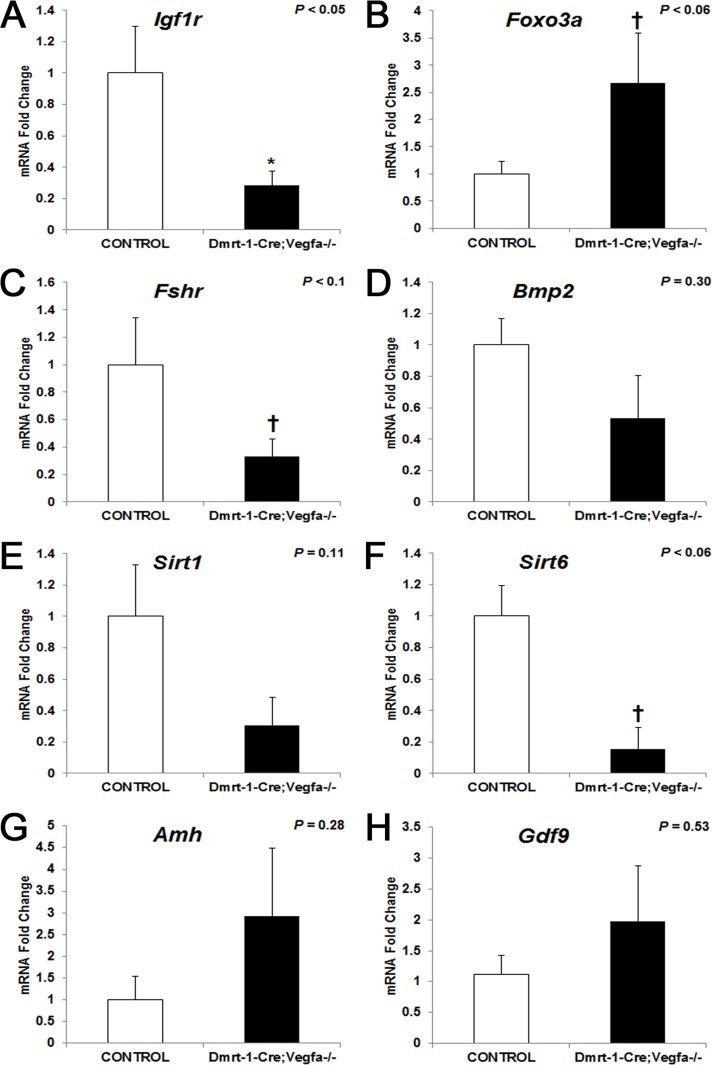
Whole ovary mRNA abundance for *Vegfa* x *pDmrt1-Cre* female mice: Igf1r (A), *Foxo3a* (B), *Fshr* (C), *Bmp2* (D), *Sirt1* (E), *Sirt6* (F), *Amh* (G) and *Gdf9* (H). Mean KO values (n = 2–6) are represented as fold changes ± SEM compared to control (n = 3–9) mean (set to 1). Results were significant when *P* < 0.05 and were represented with a *, and a † denoted data that tended to be significant (0.05 < *P* < 0.1).

Quantitative real-time PCR analysis was performed on one whole ovary from each animal to determine the expression of genes known to regulate growth and follicular development. Messenger RNA abundance for insulin-like growth factor 1 receptor (*Igfr1*) was significantly reduced 3.5-fold in ovaries lacking VEGFA isoforms (*P* < 0.05) compared to controls (Fig. 4A) at 8 months of age. Its ligand, IGF1 is important for ovarian function and proliferation of granulosa cells [19]. Forkhead boxo3a (FOXO3A) is a transcription factor that inhibits the activation of primordial follicles. Messenger RNA abundance for *Foxo3a* tended to be increased 4.8-fold in *pDmrt1-Cre;Vegfa^-/-^* ovaries (*P* < 0.06) compared to that of controls (Fig. 4B). Primordial follicles numbers were a maximum of one or two per section; thus, a difference was not detectable statistically. Follicle stimulating hormone receptor (*Fshr*) mRNA abundance tended to be reduced 3-fold in *pDmrt1-Cre;Vegfa^-/-^* ovaries (*P* < 0.1) compared to controls (Fig. 4C). FSHR is the receptor for the gonadotropin, FSH, and would be indicative of FSH signaling in the ovary. While expression of sirtuin 1 (*Sirt1*) did not differ between *pDmrt1-Cre;Vegfa^-/-^* and control ovaries (Fig. 4E), sirtuin 6 (*Sirt6*) mRNA abundance tended to be reduced 6.5-fold in *pDmrt1-Cre;Vegfa^-/-^* ovaries (*P* < 0.06) compared to control ovaries (Fig. 4F). Sirtuins are ultimately important in maintenance following caloric restriction. FOXO3A is a substrate for SIRT1, and deletion of SIRT1 results in female infertility [20–22]. There were no differences in mRNA abundance of bone morphogenetic protein 2 (*Bmp2*), anti-Müllerian hormone (*Amh*), or growth differentiation factor 9 (*Gdf9*) between KO and control ovaries (Fig. 4D, G-H) at 8 months of age. BMP family members have been shown to be regulators of the FOXOs [23]. AMH is expressed in granulosa cells prior to their differentiation and until follicles are at the early antral stage [24,25]. GDF9 is expressed by the oocyte, and when knocked out, results in impaired follicular development [26,27].

### VEGFA loss via the *Amhr2-Cre* promoter resulted in an increase in the time from mating to first parturition (three-months-of-age) and increased *Amh* mRNA abundance

**Fig 5:**
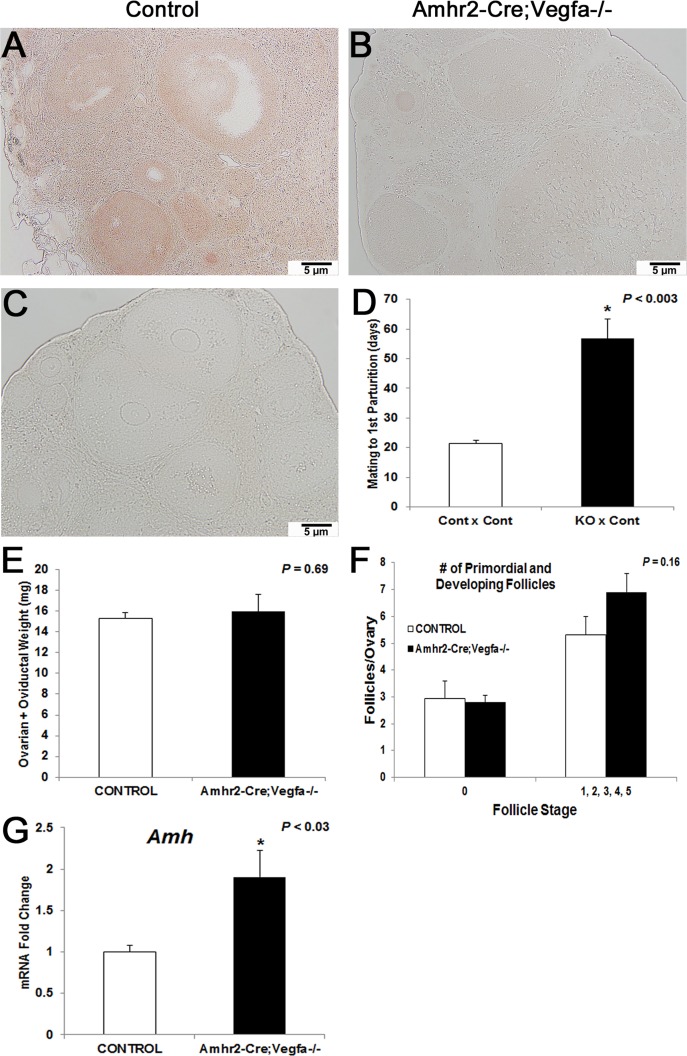
Immunohistochemistry with panVEGFA antibody to confirm VEGFA knockdown via *Amhr2-Cre*, female fertility and qPCR analysis: 200x magnification in an *Amhr2-Cre;Vegfa* control ovary (A) and an *Amhr2-Cre;Vegfa^-/-^* ovary (B). Negative control ovaries were processed without primary antibody (C). For the Control x Control and KO x Control pairs in for mating to first parturition (D), n = 4. Ovarian plus oviductal weight (mg) is presented in KO vs control females (E) while follicle counts are presented in (F) and *Amh* mRNA is represented similarly (G), and n = 10 controls and 11 KO for each graph, respectively. Results were significant when *P* < 0.05 and were represented with a *.

We confirmed the effects on fertility by elimination of VEGFA in another mouse line, *Vegfa* x *Amhr2-Cre*. These females were collected at 3-months-of-age, earlier than the *Vegfa* x *Dmrt1-Cre* females (8 months). The same pan-VEGFA antibody was used to verify knockdown of VEGFA in ovaries of *Amhr2-Cre;Vegfa^-/-^* *mice (Fig. 5B) compared to controls (Fig. 5A). Sections that were processed without primary antibody served as negative controls (Fig. 5C)*.

*On average Amhr2-Cre;Vegfa^-/-^* *mice* females took 35 days longer to get pregnant during their first mating to control males (*P* < 0.003; n = 4) than control females mated to control males in the *Vegfa* x *Amhr2-Cre* mice (Fig. 5D) with no measurable change in litter size. *Additionally, there was no difference in the combined ovarian and oviductal weight from Amhr2-Cre;Vegfa^-/-^* *(P* = 0.69) mice compared to controls (Fig. 5E) at 3 months-of-age unlike the 8-month-old *pDmrt1-Cre;Vegfa^-/-^* *females*, suggesting that the loss of VEGFA isoforms may affect fertility in a progressive manner.

While there was no difference in follicle numbers on *Amhr2-Cre;Vegfa^-/-^* *ovaries compared to controls (Fig. 5F) as well as no differences in mRNA abundance of the genes that were statistically significant in pDmrt1-Cre;Vegfa^-/-^* *ovaries (S2 Fig.), Amh* was increased 2-fold in *Amhr2-Cre;Vegfa^-/-^* ovaries (*P* < 0.03) compared to controls (Fig. 5G).

### Ovarian size was variable for *Amhr2-Cre;Vegfa^-/-^* mice

**Fig 6:**
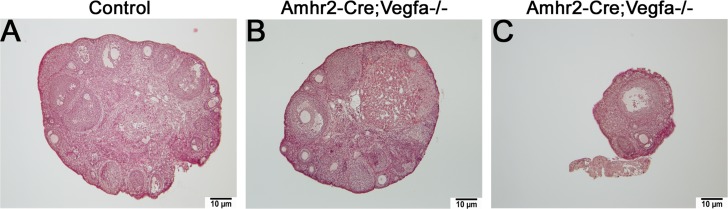
*Amhr2-Cre;Vegfa^-/-^* and control ovary histology. Hematoxylin-eosin staining of *Amhr2-Cre;Vegfa^-/-^* ovaries of variable sizes (B, C) compared to a control (A) at 100x magnification.

Ovarian size varied greatly in *Amhr2-Cre;Vegfa^-/-^* mice despite no differences in ovarian weight or follicle number. Some of the *Amhr2-Cre;Vegfa^-/-^* mice KO ovaries were similar in size to controls (Fig. 6A, B); however, some appeared greatly reduced in size (Fig. 6C). The variability in ovarian size could be due to variability in the levels of *Amhr2-Cre* [28]. The variability in ovarian size coordinated with estradiol concentrations in the plasma without overall statistical significance: low estradiol concentrations were measured in females with smaller ovaries while the high concentrations were measured in females with larger ovaries.

**Fig 7:**
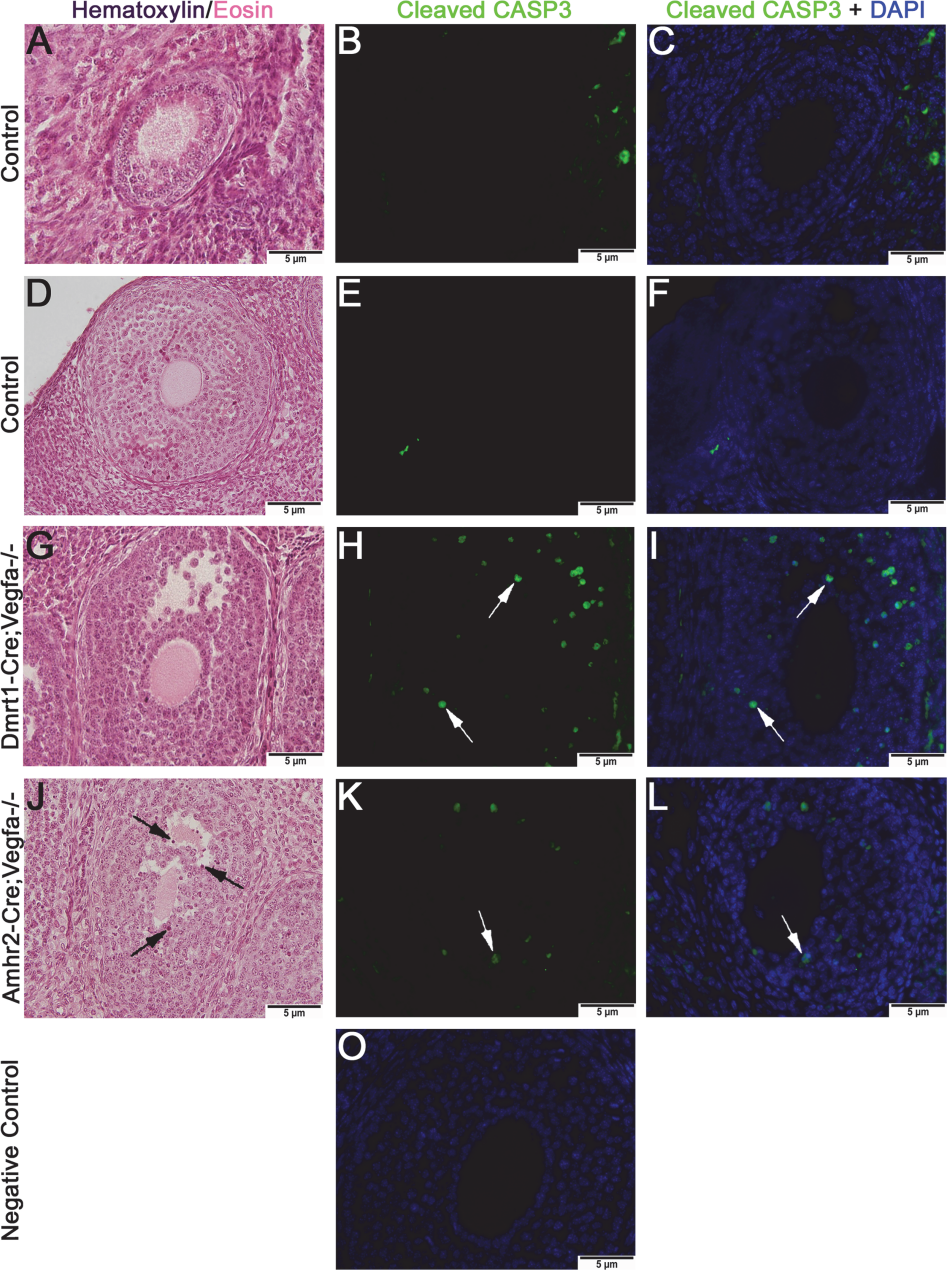
Morphology of stage 4 follicles by hematoxylin-eosin (H-E) staining and cleaved CASP3-positive staining of same follicles. H-E staining of follicles is shown at 400x for controls (A, D) and KOs (G, J). Green cleaved CASP3-positive staining is shown in follicles at 400x in controls (B, E) and in KOs (H, K). Cleaved CASP3-positive staining is shown in the same follicles but co-localized with DAPI at 400x in controls (C, F) and KOs (I,L). Negative controls were processed without primary antibody (M). Black arrows point to pyknotic granulosa cells on H-E stained follicles while arrows point to cleaved CASP3-positive granulosa cells.

**Cleaved Caspase 3-positive staining appeared more abundant in granulosa cells of *pDmrt1-Cre;Vegfa^-/-^* and *Amhr2-Cre;Vegfa^-/-^* ovaries**. Caspases are involved in apoptosis [29]. Positive staining for cleaved caspase 3 (CASP3) did not appear to be present in control ovaries (Fig. 7B-C, E-F). In both the *pDmrt1-Cre;Vegfa^-/-^* and the *Amhr2-Cre;Vegfa^-/-^* ovaries, there was cleaved CASP3-positive staining in granulosa cells not seen in controls, suggestive of granulosa cell apoptosis primarily seen in secondary follicles. Hematoxylin-eosin staining depicting the same fluorescently stained follicles is shown at 400x (Fig. 7A, D, G, J). While sections were not serial, the H-E sections were within 4–5 sections of the immunostained sections. Follicles on ovaries from *Amhr2-Cre;Vegfa^-/-^* *females exhibited granulosa cells with darker H-E staining indicative of pyknosis*.

### VE-cadherin-positive immunofluorescence did not appear different between control and *pDmrt1-Cre;Vegfa^-/-^* and *Amhr2-Cre;Vegfa^-/-^* ovaries

**Fig 8:**
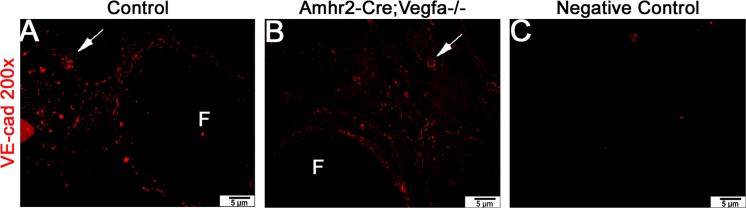
VE-cadherin-positive immunostaining in representative ovaries: *Amhr2-Cre;Vegfa^-/-^* KO ovaries (B) compared to controls from the same mouse line (A) depicted at 200x magnification. White arrows denote blood vessels. Negative controls were processed without primary antibody (C).

Ovaries from both *pDmrt1-Cre;Vegfa^-/-^* and *Amhr2-Cre;Vegfa^-/-^* mice were immunostained with an antibody to VE-cadherin, a known vascular marker. VE-cadherin-positive staining, demonstrating the presence of vasculature was at similar intensities and appeared to stain blood vessels similarly in both control and KO ovaries from both lines of mice Representative sections of *Amhr2-Cre;Vegfa^-/-^* and respective control ovaries are depicted (Fig. 8A, B). Similar results were seen in the testes of *pDmrt1-Cre;Vegfa^-/-^* males [17]. Negative controls had background reduced and were not treated with primary antibody (Fig. 8C).

## Discussion

The findings generated from the current study demonstrated that loss of VEGFA in granulosa (*pDmrt1-* and *Amhr2-Cre*) and germ cells (*pDmrt1*-Cre) resulted in subfertile females. While VEGFA has been previously implicated in fertility, this is the first time conditional loss of both pro- and antiangiogenic VEGFA isoforms has been accomplished within the granulosa cells of the ovary. The loss of VEGFA resulted in females taking longer to get pregnant, giving birth to fewer pups per litter, having reduced plasma estradiol, containing more apoptotic granulosa cells, resulting in fewer developing follicles and a tendency to have fewer corpora lutea. Additionally, the subfertility phenotype was supported in *Amhr2-Cre;Vegfa^-/-^* *ovaries (3 months of age) and appeared more pronounced in the pDmrt1-Cre;Vegfa^-/-^* *mice which was presumably due to the later age of these mice at collection (8 months of age); however, we cannot ignore that the differences between the mouse lines could also be due to the different cell types suffering VEGFA isoform loss*. This is currently being investigated in our laboratory through a granulosa cell-specific elimination of VEGFA. The phenotypes from both of these mouse lines support earlier findings in our laboratory regarding the importance of VEGFA isoforms in follicle recruitment and development [6,7]. Furthermore, in mice overexpressing antiangiogenic VEGFA165b that were collected between 2- and 4-months-of-age, there is a similar phenotype with half the litter size, fewer mature follicles, and fewer CL [30]. Thus, eliminating all VEGFA isoforms or overexpression of VEGFA165b in granulosa cells appears to reduce female fertility.

Both *pDmrt1-Cre;Vegfa^-/-^* (8-months-old) and *Amhr2-Cre;Vegfa^-/-^* females (3-months-old) were subfertile. The longer period leading to parturition in female KO mice compared to controls suggested a decreased rate of ovulation in the female KO mice or ovulation of oocytes that are not competent to be fertilized. Ha *et al*., showed that estrogen administration increases ovarian VEGFA expression and suggested that VEGFA improves the number and quality of oocytes [31]. In our study, the loss of VEGFA in *pDmrt1-Cre;Vegfa^-/-^* resulted in reduced estrogen in blood plasma which was presumably due to a reduced number of follicles developing. As mentioned previously, ovarian weight and plasma estradiol concentrations had a positive relationship in *Vegfa* x *Amhr2-Cre* females. The reduction in estrogen levels may also contribute to a reduction in the quality of oocytes. The aromatase knockout mouse, the estrogen β receptor knockout mouse, and the estrogen α receptor knockout mouse have demonstrated that estrogen enhances the number of follicles progressing to the early antral stage [32–35]. Furthermore, a positive relationship between VEGFA and E_2_ concentrations in gilt follicular fluid suggests a possible cause-and-effect relationship [36] with VEGFA stimulating estrogen production by increasing vascular permeability to cholesterol and increasing signal transduction pathways activating enzymes that are critical for estrogen production. One study showed that plasma 17β-estradiol concentration in rats treated with VEGFA120 and 164 were significantly higher than that in the control rats, indicating that VEGFA120 and 164 may indirectly stimulate the production of 17β-estradiol via granulosa cells [37].

In our study, plasma 17β-estradiol levels in the *pDmrt1-Cre;Vegfa^-/-^* were significantly lower than that in the controls. Our results indicated that loss of VEGFA in granulosa cells inhibits the production of 17β-estradiol in *pDmrt1-Cre;Vegfa^-/-^* and the granulosa cells from both *pDmrt1-Cre;Vegfa^-/-^* and *Amhr2-Cre;Vegfa^-/-^* mice had increased staining for cleaved CASP3 as well as an increased number of pyknotic nuclei in granulosa cells. Previous studies have demonstrated that intrabursal injections of VEGFA trap (an inhibitor of VEGFA) resulted in reduced granulosa cell proliferation [38]. Thus, the *in vivo* phenotype of both of our KO mouse lines supports a role for VEGFA in granulosa cell survival and in enhancing its ability to produce steroid hormones such as estradiol promoting the viability of follicle development and progression.

McFee *et al*. previously demonstrated a role for VEGFA in primordial follicle activation, maturation and survival [6]. Rat ovaries treated transiently with VEGFA_164 were composed of fewer primordial follicles (stage 0) and more developing follicles (stages 1–4) than controls. Ovaries treated with VEGFAxxxB antibody to neutralize all antiangiogenic isoforms had fewer primordial and early primary follicles and more primary, transitional, and secondary follicles compared to controls (7). These data indicate that VEGFA proangiogenic isoforms promote follicle development, and in contrast, VEGFA antiangiogenic isoforms maintain primordial follicles in an arrested state. Removal of VEGFA antiangiogenic isoforms allows for increased progression of primordial follicles to the developing follicle pool [7]. Given previous findings in our laboratory with transient treatments in culture, we suspect that the number of primordial follicles is increased in the *pDmrt1-Cre;Vegfa^-/-^* females’ ovaries given the increase in *Foxo3a* mRNA even if differences were not detectable. At the same time, the differences may not be statistically relevant given the apparent increase in cleaved CASP3-positive granulosa cells, suggesting that some of the follicles that did advance in the *pDmrt1-Cre;Vegfa^-/-^* females regressed prematurely. VEGFA165 also promoted the primary-to-secondary follicle transition in bovine ovaries activated *in vitro* [39]. Furthermore, *in vivo* studies showed that inhibition of VEGFA and KDR can inhibit follicular development or prevent ovulation [40,41]. Thus, in addition to follicular development VEGFA also appears to affect ovulation in later stage preovulatory follicles.

In our study, ovarian weight in KO mice was reduced compared to that of control females. This suggests that loss of VEGFA in granulosa and germ cells within mouse ovaries affects ovarian size through reductions of follicle development and/or maturation. Another reason for the reduced ovarian size in the KO females is due to the reduced number of CL despite no change in progesterone. An *in vivo* study showed that the VEGFA angiogenic isoform promoted ovarian follicular angiogenesis leading to stimulatory effects on subsequent follicular development and numbers of oocytes ovulated in mature cycling rats [37]. Furthermore, inhibition of VEGFA and KDR suppressed follicular development and ovulation [40,41]. Presumably, the fewer CL in ovaries of *pDmrt1-Cre;Vegfa^-/-^* females may be caused by fewer antral follicles and subsequently fewer ovulations in those mice, ultimately resulting in smaller ovaries and a marked reduction in litter size compared to controls.

Another plausible theory for the reduction in ovarian weight in the KO females would be that loss of VEGFA in granulosa cells may reduce angiogenesis, the blood supply for oxygen, nutrients and influence the growth of the ovary and follicle maturation. However, this model utilizes the Cre-lox approach to eliminate VEGFA from granulosa cells and oocytes, cells that are avascular. Still, we investigated ovarian vasculature via immunostaining. Ovarian vasculature in two similar KO mouse models (*pDmrt1-Cre;Vegfa^-/-^* and *Amhr2-Cre;Vegfa^-/-^*) did not appear different from controls based on positive staining of VE-cadherin. Similarly, we did not see effects in *pDmrt1-Cre;Vegfa^-/-^* male mice where VEGFA was eliminated in Sertoli and germ cells [17].

To further characterize the reproductive phenotypes caused by VEGFA loss, we performed qPCR analysis of genes that are known regulators of ovarian function. FOXO3A is a key oocyte factor critical for suppressing primordial follicle activation [22]. Liu *et al*. reported that loss of *Foxo3a* expression in mice results in premature follicle activation [42]. *Foxo3a* mRNA was 4.8-fold higher in KO ovaries than controls, suggestive of potential suppression of follicle progression in these ovaries. Moreover, members of the FOXO family have been shown to regulate many vital cellular processes including metabolism [43,44]. Interestingly, it has been suggested that insulin and IGF-1 signaling inactivate the FOXOs in other tissues [45]. FOXO transcription factors are further implicated in this study by the 3.5-fold decrease in *Igf1r* mRNA in the ovaries of the females following VEGFA loss. Additionally, IGF1R expression has been demonstrated to be highest in large luteal cells of the bovine CL; thus, the disruption in follicle progression in our mice and subsequent reduction in CL number could be a result of the reduction in *Igf1r* [46] since whole ovaries were analyzed. It has also been demonstrated that FSH and IGF1 work synergistically in human, mouse and rat granulosa cells to stimulate AKT-dependent granulosa cell proliferation [47]. These findings, together with our observed reductions in mRNA abundance for *Igf1r* and *Fshr*, further support the apoptotic granulosa cells seen in ovaries of both *pDmrt1-Cre;Vegfa^-/-^* and *Amhr2-Cre;Vegfa^-/-^* mice as well as the reduction in litter size of *pDmrt1-Cre;Vegfa^-/-^* *females*. Also striking is the 6.5-fold reduction in *Sirt6* because of the direct relationship of sirtuins with FOXO3A and infertility [48]. It has been posited that AMH increases VEGFA; thus, the increase in *Amh* mRNA abundance in *Amhr2-Cre;Vegfa^-/-^* ovaries could be to atone for the elimination of VEGFA in follicular cells [49]. While we did not detect differences in follicle counts, ovarian weight or plasma estradiol concentrations when *Vegfa* was lost via the *Amhr2-Cre* promoter, we did see prolonged first mating period and variability in ovarian size as well as the presence of apoptotic granulosa cells. Thus, the increase in *Amh* mRNA could also be explained to be a block against the follicle’s sensitivity to FSH [49]. Again, these mice were harvested at 3-months-of-age, and the phenotype did not appear as progressed as in the *pDmrt1-Cre;Vegfa^-/-^* *mice at 8 months of age*.

In conclusion, we demonstrated that removal of VEGFA angiogenic and antiangiogenic isoforms in granulosa cells via the *pDmrt1* promoter resulted in reduced ovarian weight, fewer CL and reduced estrogen concentrations (by 50%). Furthermore, the time period between mating and parturition was longer in both *pDmrt1-Cre;Vegfa^-/-^* and *Amhr2-Cre;Vegfa^-/-^* mice than in controls, and *pDmrt1-Cre;Vegfa^-/-^* females gave birth to fewer pups. Additionally, *pDmrt1-Cre;Vegfa^-/-^* females had increased *Foxo3a*, reduced *Igf1r, Fshr*, and *Sirt6* mRNA levels. These data are suggestive of primordial follicle arrest and reduced proliferation, further implicated by increased apoptotic granulosa cells as indicated by immunofluorescence that was observed in both *pDmrt1-Cre;Vegfa^-/-^* and *Amhr2-Cre;Vegfa^-/-^* mice. The present data suggest that VEGFA is necessary for normal follicular development, estrogen synthesis, and ovulation. These reductions in ovarian function likely contributed to the increased interval from mating to parturition in both *pDmrt1-Cre;Vegfa^-/-^* and *Amhr2-Cre;Vegfa^-/-^* mice. Ultimately, these data support the concept that the loss of VEGFA results in subfertility by limiting follicle progression and subsequent ovulation and that this phenotype appears to be more dramatic as the female progress in age as demonstrated by the 3-month *Amhr2-Cre;Vegfa^-/-^* and 8-month *pDmrt1-Cre;Vegfa^-/-^* phenotypes or due to differences in cell-specific VEGFA loss.

## Supporting Information

S1 Fig Schematic depicting conditional elimination of VEGFA isoforms occurs in granulosa cells. (PPTX)

S2 Fig Whole ovary mRNA abundance for *Vegfa* x *Amhr2-Cre* female mice: *Igf1r* (A), *Foxo3a* (B), *Fshr* (C), *Sirt6* (D), and *Gdf9* (E). Mean KO values (n = 3–5) are represented as fold changes ± SEM compared to control (n = 4) mean (set to 1).
(TIF)
