# Supplementary figures and images for: Loss of Vascular Endothelial Growth Factor A (VEGFA) Isoforms in Granulosa Cells Using pDmrt-1-Cre or Amhr2-Cre Reduces Fertility by Arresting Follicular Development and by Reducing Litter Size in Female Mice
Source: PLoS One. 2015 Feb 6;10(2):e0116332. doi: 10.1371/journal.pone.0116332 (PMC4320103; doi:10.1371/journal.pone.0116332)

## Slide 1
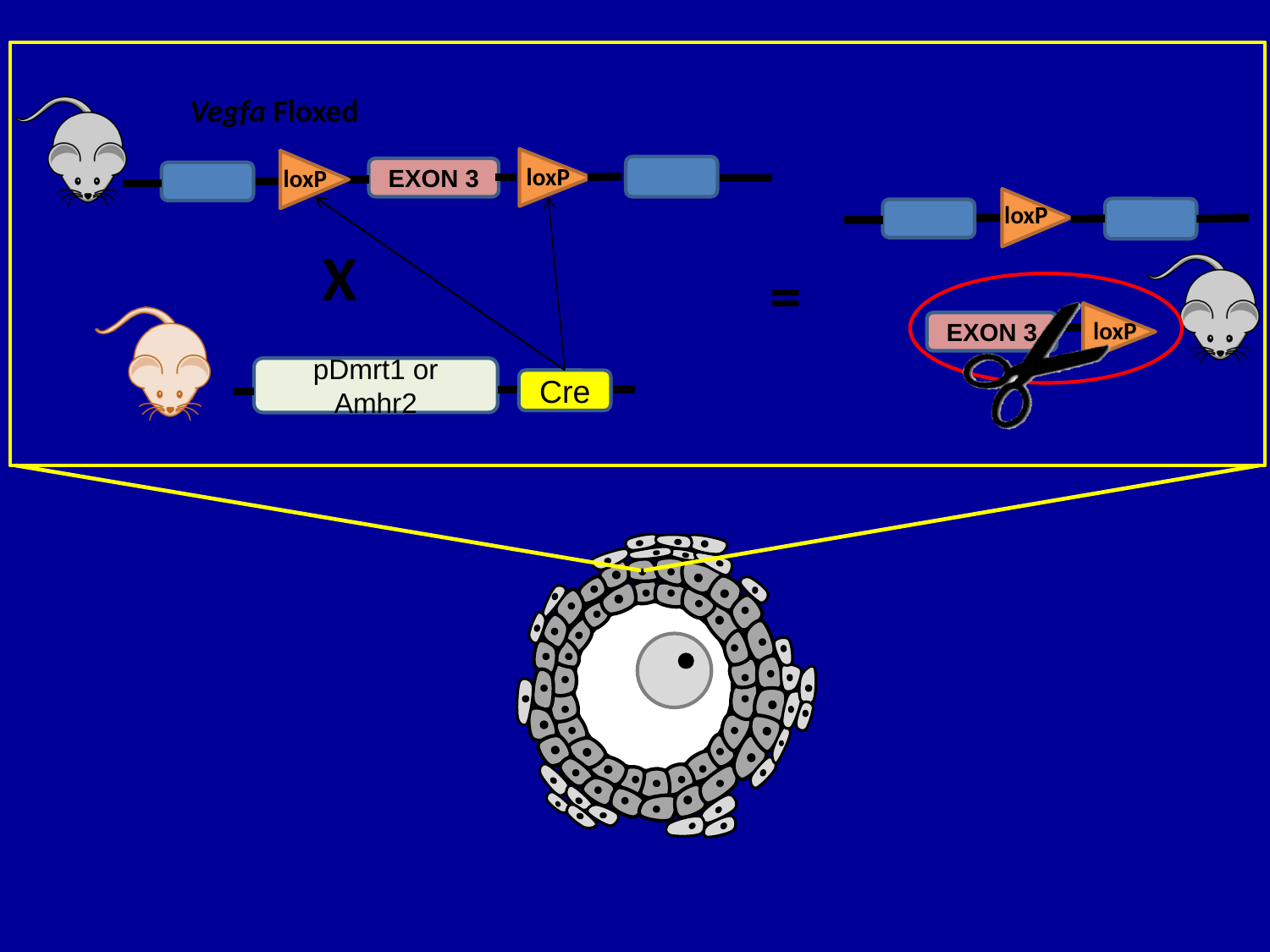

Vegfa Floxed
loxP
loxP
EXON 3
loxP
X
loxP
EXON 3
=
pDmrt1 or Amhr2
Cre

Supplement: S1 Fig — (PPTX) [file pone.0116332.s001.pptx]

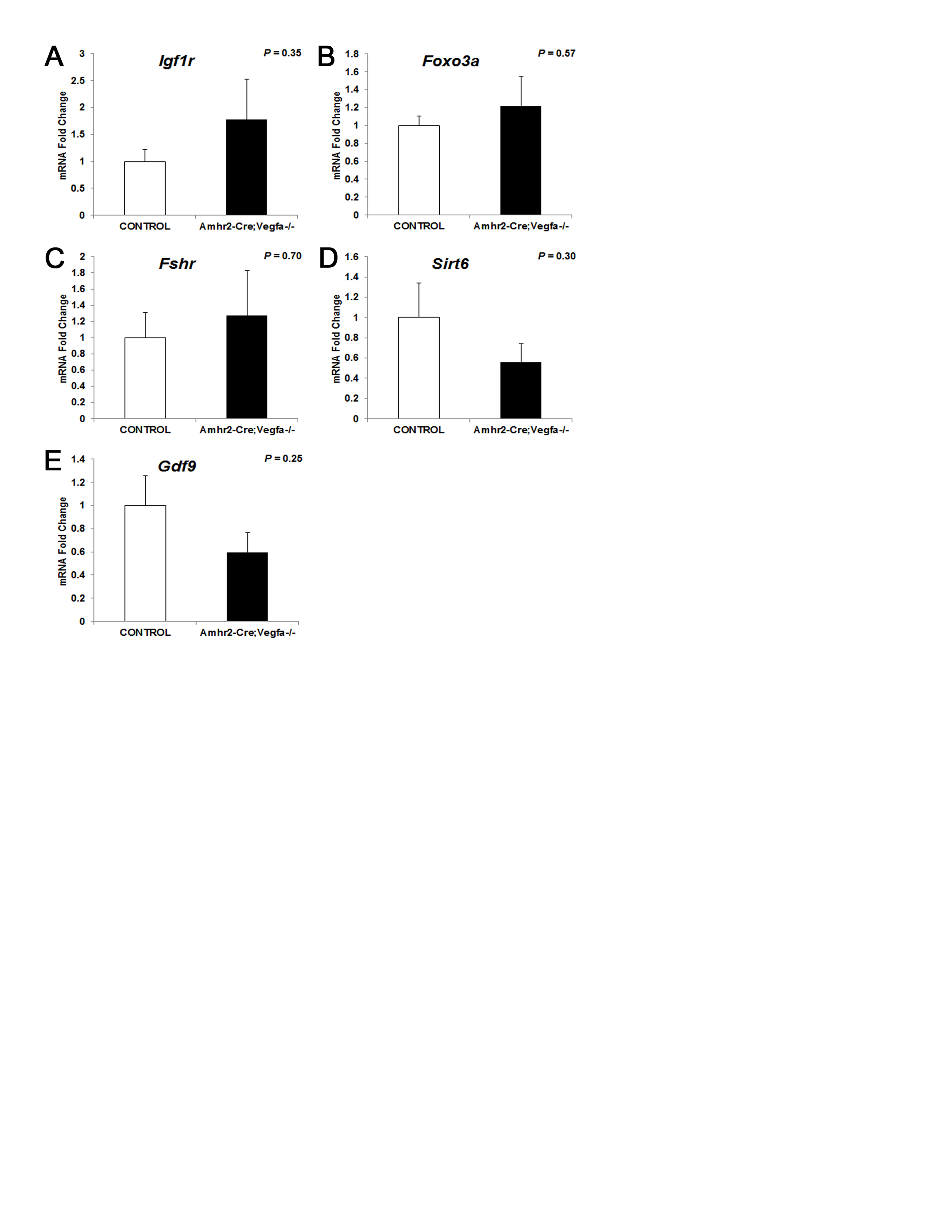

Supplement: S2 Fig — Mean KO values (n = 3–5) are represented as fold changes ± SEM compared to control (n = 4) mean (set to 1). (TIF) [file pone.0116332.s002.tif]
